# The Processed Amino-Terminal Fragment of Human TLR7 Acts as a Chaperone To Direct Human TLR7 into Endosomes

**DOI:** 10.4049/jimmunol.1402703

**Published:** 2015-04-27

**Authors:** Madeleine M. Hipp, Dawn Shepherd, Sarah Booth, Dominic Waithe, Caetano Reis e Sousa, Vincenzo Cerundolo

**Affiliations:** * MRC Human Immunology Unit, MRC Weatherall Institute of Molecular Medicine, Radcliffe Department of Medicine, University of Oxford, Oxford OX3 9DU, United Kingdom; and; † The Francis Crick Institute, Lincoln's Inn Fields Laboratory, London WC2A 3LY, United Kingdom

## Abstract

TLR7 mediates innate immune responses to viral RNA in endocytic compartments. Mouse and human (h)TLR7 undergo proteolytic cleavage, resulting in the generation of a C-terminal fragment that accumulates in endosomes and associates with the signaling adaptor MyD88 upon receptor triggering by TLR7 agonists. Although mouse TLR7 is cleaved in endosomes by acidic proteases, hTLR7 processing can occur at neutral pH throughout the secretory pathway through the activity of furin-like proprotein convertases. However, the mechanisms by which cleaved hTLR7 reaches the endosomal compartment remain unclear. In this study, we demonstrate that, after hTLR7 proteolytic processing, the liberated amino (N)-terminal fragment remains bound to the C terminus through disulfide bonds and provides key trafficking information that ensures correct delivery of the complex to endosomal compartments. In the absence of the N-terminal fragment, the C-terminal fragment is redirected to the cell surface, where it is functionally inactive. Our data reveal a novel role for the N terminus of hTLR7 as a molecular chaperone that provides processed hTLR7 with the correct targeting instructions to reach the endosomal compartment, hence ensuring its biological activity and preventing inadvertent cell surface responses to self-RNA.

## Introduction

Toll-like receptors are important components of the innate immune system, serving to alert the host to viral or microbial invasion. Many TLRs recognize pathogen-associated molecular patterns that are the end product of microbe-restricted biosynthetic pathways and, therefore, are qualitatively distinct from host components (1). In contrast, TLRs 7, 8, and 9 recognize viral or bacterial RNA or DNA molecules that are highly homologous to those of the host (2–5). Hence, the ability of these TLRs to discriminate between self and nonself nucleic acids was proposed to occur through spatial segregation. Indeed, all nucleic acid–binding TLRs respond to their agonist ligands within endolysosomal compartments, which commonly serve as virus entry ports but are not accessed under physiological conditions by self RNA or DNA (6, 7). Nevertheless, targeting self RNA or DNA to endosomes can trigger activation of nucleic acid–binding TLRs (3, 4), and certain autoimmune disorders, such as systemic lupus erythematosus, are associated with TLR7 and/or TLR9 activation (8, 9).

Nucleic acid–binding TLRs are made as proproteins that are proteolytically processed. Processing releases an N-terminal fragment away from the membrane-anchored C terminus, which is then competent to respond to agonist ligands. The requirement for processing is thought to help restrict receptor activation to endolysosomes, because these acidic compartments are rich in low pH–dependent proteases, such as cathepsins (10, 11) and asparaginyl endopeptidase (12, 13), which are able to process human (h)TLR9 and mouse (m)TLR9, as well as mTLR7. However, we showed recently that low pH is not required for the processing of hTLR7, which can be mediated by furin-like protein convertases that function at neutral pH (14). This family of proteases is present throughout the secretory pathway and, therefore, is able to cleave its substrates in the endoplasmic reticulum (ER) (15), *trans*-Golgi network, or at the cell surface (16, 17). This suggests that processing within endosomes is not always necessary to localize receptor activity to those compartments and raises the possibility that some TLRs, such as hTLR7, may be processed early in the biosynthetic pathway. However, a genetically truncated form of hTLR7 corresponding to the processed C-terminal fragment fails to confer responsiveness to TLR7 agonists (14). These results raise the question of how hTLR7 can traffic from the ER to endosomes for functional activity if it is naturally processed prior to arrival at its destination.

In this article, we show that the C-terminal fragment of hTLR7, when expressed on its own, is directed to the cell surface where is it is incapable of mediating responses to agonist ligands. Coexpression of the amino N-terminal fragment leads to disulfide bond–mediated *trans*-association with the C terminus and redirects the latter to endosomes, restoring receptor function. Thus, the N-terminal fragment of hTLR7 is necessary for receptor delivery to endosomes, and its ability to remain bound to the C-terminal fragment ensures delivery of the signaling-competent receptor to the site of ligand recognition, even if receptor processing occurs early during biosynthesis.

## Materials and Methods

### Reagents

Imiquimod (R837) was from InvivoGen. LPS (*Escherichia coli* type), anti-FLAG, PMA, anti-mouse agarose, anti-HA (clone HA-7) agarose, anti-FLAG (M2) agarose, HA peptide, and 3×FLAG peptide were from Sigma-Aldrich. Monoclonal anti-HA 3F10 and complete protease inhibitor tablets were from Roche. Anti-human CD107A (Lamp1) was from AbD Serotec. Anti-calnexin C-20 was from Santa Cruz Biotechnology, and anti-EEA1 was from Cell Signaling Technology. Anti-rat HRP, anti-mouse HRP, and anti-goat HRP were from R&D Systems, and anti-IL-8–allophycocyanin was from BD Pharmingen. Anti-rabbit Alexa Fluor 555, anti-mouse Alexa Fluor 647, and streptavidin-coated Dynabeads were from Invitrogen. Goat anti-mouse Abberior STAR 440SX-conjugated Ab was a kind gift from Christian Eggeling (University of Oxford). For hIL-8 ELISA, purified anti–hIL-8, monoclonal (clone G265-5; #554716) and biotinylated anti–hIL-8, monoclonal (#554718) were used. The cell surface biotinylation kit was from Pierce Thermo Scientific. The QuikChange II XL Site-Directed Mutagenesis Kit was from Agilent Technologies.

### Cell culture media, cell lines, and PMA differentiation

RPMI 1640 supplemented with 10% (v/v) heat-inactivated FCS, penicillin/streptomycin, l-glutamine, 25 mM HEPES, nonessential amino acids, 1 mM sodium pyruvate, 50 μM 2-ME, and NaHCO_3_ (Sigma) (hereafter referred to as R10). DMEM high glucose supplemented with 10% (v/v) heat-inactivated FCS, penicillin/streptomycin, and l-glutamine (hereafter refereed to as D10). Human THP-1 cells (myelomonocytic) were cultured in R10, and HEK293T cells were cultured in D10. Cells were grown at 37°C in humidified air with 5% CO_2_. Unless indicated otherwise, monocytic THP-1 cells were differentiated into a macrophage-like cell line by culturing them in R10 supplemented with 10–15 nM PMA for 24–72 h.

### DNA cloning, site-directed mutagenesis, and lentiviral transduction

Human *Tlr7* and preprocessed *Tlr7* C-terminal fragment tagged at the C terminus with HA, referred to as 7 and 7C, respectively, were cloned into the enhanced GFP-expressing HIV-based lentiviral vector pHR-SIN-IRES-Em (18), as described previously; the 7C fragment corresponds to the previously described CtermK fragment (14) (Supplemental Table I). Additional C-terminal fragments–CtermG, CtermH, CtermI, and CtermJ–were cloned as previously described (14). The N terminus FLAG and C terminus HA-tagged *Tlr7*, referred to as F-7, was generated by the addition of the sequence DYKDDDDK directly after the predicted leader sequence. Briefly, two PCR amplifications were performed using the lentiviral vector coding for 7 as a template. A short 122-bp fragment was generated using primers BclIKozak forward (Fwd) and Flag reverse (Rev), and a larger 3.1-kb fragment was generated using primers Flag Fwd and Rev hTLR7HA. These fragments were annealed together via their overlapping FLAG sequence and amplified using PCR and primers BclIKozak Fwd and Rev hTLR7HA. The resulting cDNA was inserted into the parental BamHI/XhoI-flanked pHR-SIN-IRES-Em lentiviral vector. The N-terminally FLAG-tagged Cterm fragment (F-7C) was created by PCR amplification from pHR-SIN-CtermKHA-IRES-Em (14) using primers CtermK Flag Fwd and Rev hTLR7HA. The FLAG-tagged CtermK cDNA was inserted into the parental BamHI/XhoI-flanked pHR-SIN-IRES-Em lentiviral vector. FLAG-tagged N-terminal constructs were generated by fusing the N-terminal cDNA of *Tlr7* to a 587-nt IRES sequence and inserting into the pHR-SIN-mCherry lentiviral vector. Two N-terminal TLR7 fragments were engineered: a longer fragment corresponding to aa 1–476 of h*Tlr7* (F-7N) and a shorter version corresponding to aa 1–467. Briefly, the N-terminal fragments were amplified using PCR and primers TLR7 mlu1 Fwd and TLR7 Nterm Rev from the lentiviral plasmid coding for F-7. The IRES sequence was amplified using PCR and primers IRES Fwd and IRES Rev from the parental pHR-SIN-IRES-Em plasmid. The two DNA fragments were cut with Bcl1 and ligated together. The resulting product was amplified using PCR and primers TLR7 mlu1 Fwd and IRES Rev and inserted into the parental MluI/BamHI-flanked pHR-SIN-mCherry plasmid to produce pHR-SIN-FlagNTermTLR7-IRES-mCherry constructs. A version of the longer N-terminal hTLR7 without a FLAG tag was made in a similar way using hTLR7 as a template. The TLR7/TLR4 chimera was constructed by “PCR sewing” of cDNA corresponding to aa 1–833 of hTLR7 and aa 628–850 of hTLR4-HA. “PCR sewing” was achieved by multiple PCRs using primers with overlapping sequences of TLR7 and TLR4 (see below). Briefly, the ectodomain of hTLR7 [TLR7(Ecto)] was amplified using PCR and primers Fwd hTLR7HA and A hTLR7/4 PCR Rev from the pUNO-hTLR7-HA (InvivoGen) plasmid, generating a TLR7(Ecto) fragment fused C terminally to a small part of TLR4-cDNA. Transmembrane and cytoplasmic domains of TLR4 were amplified using PCR and primers A hTLR7/4 PCR Fwd and mTLR7HA PCR Rev from the pUNO-hTLR4A-HA plasmid (InvivoGen), generating a TLR4(TM+CP) fragment with a small part of TLR7-cDNA fused to its N terminus. Finally, fragments TLR7(Ecto) and TLR4(TM+CP) were mixed at a 1:1 ratio and hybridized; this served as a template for the third PCR using primers Fwd hTLR7HA and mTLR7HA PCR Rev. The resulting cDNA was cloned into the parental BamHI/XhoI-flanked pHR-SIN-IRES-Em lentiviral vector. C-terminal Ist2-tagged TLR7 (TLR7-Ist2) was constructed by fusing cDNA encoding the full-length TLR7 to cDNA encoding the last 69 aa of yeast Ist2p via a short linker sequence ([Gly-Gly-Gly-Gly-Ser]×3). cDNA coding for the Ist2p fragment was synthesized by GeneArt. Briefly, cDNA encoding the last 69 aa of yeast Ist2p was amplified using PCR and primers Ist2 lenti Fwd and Ist2 lenti Rev. The reverse primer contains an overhang coding for the HA epitope. YFP from a previously cloned pHR-hTLR7-YFP plasmid was replaced through the Ist2-HA fragment. The resulting plasmid was used as a template to amplify hTLR7-Ist2-HA using PCR and primers TLR7-Ist2 Bcl1 Fwd and TLR7-Ist2 SalI Rev. The PCR product was ligated with pHR-SIN-IRES-Em. Mutant versions of hTLR7-HA were produced from the original lentiviral vector using a site-directed mutagenesis kit, according to the manufacturer’s instructions. To produce lentiviral particles, HEK293T cells were cotransfected with plasmids encoding VSV-G and Gag-Pol (19), as well as the appropriate lentiviral vectors using FuGENE 6 (Roche), per the manufacturer’s instructions. Medium containing viral particles was collected 48 and 72 h after transfection and added directly to cells for infection. Alternatively, viral particles were concentrated by sucrose gradient density centrifugation (JA-25.50 rotor, 24000 rpm, 4°C, 2 h) and then added to cells. The day after transduction, cells were given fresh medium and cultured. After expanding, GFP^+^ and/or mCherry^+^ cells were isolated by FACS sorting.

### Cell lysis and quantitative immunoblot analysis

One million cells were lysed in 50 μl lysis buffer consisting of 20 mM Tris-Cl (pH 7.5), 150 mM NaCl, 1 mM EDTA (pH 8), 1% (v/v) Triton X-100, 1 mM EGTA, 2.5 mM sodium pyrophosphate decahydrate, and 1 mM β-glycerophosphate supplemented with complete protease inhibitors (Roche). Proteins were separated by SDS-PAGE, transferred to polyvinylidene difluoride membranes, blocked for 1 h with 5% (w/v) skim milk in PBS with 0.5% (v/v) Tween 20, and probed for 1 h with the appropriate Abs. Membranes were washed three times with PBS with 0.5% (v/v) Tween 20 and incubated for 1 h with HRP-conjugated secondary Ab. After washing, proteins were visualized with an ECL detection reagent (Pierce).

### Immunoprecipitation

PMA-differentiated cells were lysed as described above. Cell lysate was precleared for 1 h at 4°C with mIgG-Agarose. For immunoprecipitation, samples were incubated for 2 h at 4°C with anti-HA–Agarose or anti-FLAG–Agarose. Agarose was washed four times with ice-cold coimmunoprecipitation buffer (50 mM Tris-Cl [pH 7.5], 1 mM EDTA, 150 mM NaCl, and 0.5% Nonidet P-40), and incubating agarose with 200 μg/ml HA Peptide or 3X FLAG Peptide then eluted bound proteins.

### Cell surface biotinylation

A total of 4 × 10^7^ THP-1 cells stably expressing GFP, TLR7, 7C, or TLR7/4 was seeded in 80 ml R10 and either differentiated with 50–200 U/ml hIFN-γ for 24 h or left untreated. After washing twice in cold PBS, cell surface biotinylation was performed using the Cell Surface Isolation Kit from Pierce Thermo Scientific, according to the protocol of the manufacturer. Briefly, cells were incubated for 30 min on ice on a rocking platform, with or without 0.25 mg/ml Sulfo-NHS-SS-Biotin (1 × 10^7^ cells/ml; Pierce). GFP-expressing cells served as a control. To quench any free biotin, 50 μl Quenching Solution (Pierce Thermo Scientific) was added per milliliter of cell suspension, and cells were washed once with TBS. Cells were lysed and biotinylated; proteins were immunoprecipitated using an immobilized NeutrAvidin gel (Pierce Thermo Scientific) or using streptavidin-coated Dynabeads (Invitrogen), according to the manufacturer’s protocol. Immunoprecipitated proteins were eluted by boiling in 1× denaturing protein loading buffer for 5 min at 95°C.

### IL-8 assay

Cells were stimulated for 24 h with the indicated TLR agonists. Conditioned medium was collected, and secretion of hIL-8 was analyzed by ELISA.

### Intracellular staining for IL-8

PMA-differentiated THP-1 cells were stimulated with the indicated agonists. Thirty minutes after stimulation, GolgiPlug (BD Bioscience) was added for ≤12 h, and cells were fixed, permeabilized, blocked, and stained with anti-IL-8–allophycocyanin or the corresponding isotype control, according to the manufacturer’s protocol (eBioscience).

### FACS cell surface and intracellular staining

A total of 1 × 10^5^ THP-1 cells was seeded per well of a 96-well flat-bottom plate in 200 μl R10 containing 15 nM PMA and incubated for 3 d. Cells were harvested, and Aqua (Molecular Probes) was used to stain dead cells, according to the manufacturer’s procedure. Cells were fixed with Fixation Buffer (eBioscience) for 20 min at room temperature. After washing, cells were permeabilized in permeabilization buffer (eBioscience) for intracellular staining or were directly incubated in blocking buffer (PBS + 5% heat-inactivated human serum + 5% FCS + 10% heat-inactivated goat serum [Sigma] + 1:20 diluted Human Fc Receptor Binding Inhibitor [eBioscience]; sterile filtered) for cell surface staining, and then were stained with anti-FLAG or anti-HA primary Ab, followed by anti-mouse or anti-rabbit Alexa Fluor 647 secondary Ab. For intracellular staining, blocking buffer and Ab-staining solutions were made up in permeabilization buffer instead of in PBS. Cells were acquired with a Fortessa cell analyzer (BD).

### Phagosome isolation

Forty million THP-1 cells stably expressing TLR7-HA were differentiated with 10 nM PMA for 24 h. The following day, cells were pulsed for 45 min at 37°C with 80 μl 2-μm latex beads (Polybead Polystyrene Microspheres; Polysciences). Beads were washed away by rinsing twice with ice-cold PBS and incubated for 2–6 h at 37°C. After rigorous washing in ice-cold PBS, THP-1 cells were scraped into 3 ml sucrose homogenization buffer (250 mM sucrose and 3 mM imidazole in water) and pelleted. Cells were resuspended in 1 ml sucrose homogenization buffer plus protease inhibitor mixture (Roche) and EDTA and disrupted by 20 strokes in a dounce homogenizer. The disrupted cells were rocked gently for 10 min at 4°C to free the intracellular contents and centrifuged at 8000 × *g* to remove intact cells, nuclei, and debris. A total of 50 μl of the supernatant was set aside as pregradient control and lysed by adding 1% Triton X-100 and protease inhibitors. To separate intact phagosomes from the remaining supernatant, the supernatant was mixed with 1 ml 60% sucrose and applied to an ice-cold SW 40 tube (Ultra-Clear centrifuge tubes, 14 × 89 mm; Beckman Coulter) stacked with a sucrose gradient as follows: 1 ml 60% sucrose, 2 ml homogenate, 2 ml 32% sucrose, 2 ml 20% sucrose, and 2 ml 10% sucrose. The gradient was spun at 100,000 × *g* for 65 min in a Beckman centrifuge with an SW 41 Ti rotor at 4°C. Following centrifugation, phagosomes were harvested from the 20–10% sucrose interface. Phagosomes were transferred to a clean SW 40 tube, diluted with ice-cold PBS to a final volume of 12 ml, and spun at 40,000 × *g* for 15 min at 4°C in an SW 41 Ti rotor. The phagosome pellet was lysed in lysis buffer. For Western blot analysis, loading of phagosome samples was normalized either by volume or by the number of phagocytosed beads.

### Confocal microscopy

THP-1 cells were cultured on poly-l-lysine–coated glass slides in the presence of 10 nM PMA for 24 h at 37°C. Samples were fixed with 4% paraformaldehyde (Sigma) for 15 min at room temperature and permeabilized twice for 1 min with 0.5% Triton-X 100. Blocking buffer (PBS containing 5% human serum [PAA], 5% FCS, 1% BSA, 5% goat serum, and 1:20 diluted Human Fc Receptor Binding Inhibitor [eBioscience]) was added for 1–2 h before samples were treated with primary Abs in blocking buffer for 30 min. Samples were washed three times with PBS and stained with secondary Abs in blocking buffer for 30 min, washed again as before, and mounted using Fluoromount, with or without DAPI (SouthernBiotech). All images were taken to avoid saturation using a confocal microscope (Zeiss 780; Carl Zeiss) equipped with a chromatically corrected Apochromat 63× lens. This lens is designed to minimize chromatic aberration, which makes it ideal for colocalization studies. Analysis was done on raw image data. For quantitative colocalization analysis, Li’s coefficient was calculated using the application Coloc2 in Fiji (20) (http://imagej.nih.gov/ij/). Such analysis allows characterization of the degree of overlap between two channels in the multidimensional microscopy image recorded at different emission wavelengths.

### Statistics

All data are shown as mean ± SD. An unpaired two-way Student *t* test or two-way ANOVA was performed to determine the difference between the control and treated groups. Significance was accepted at *p* < 0.05 versus control.

## Results

### The N-terminal hTLR7 fragment associates with and restores functional activity of the processed hTLR7

**FIGURE 1.**
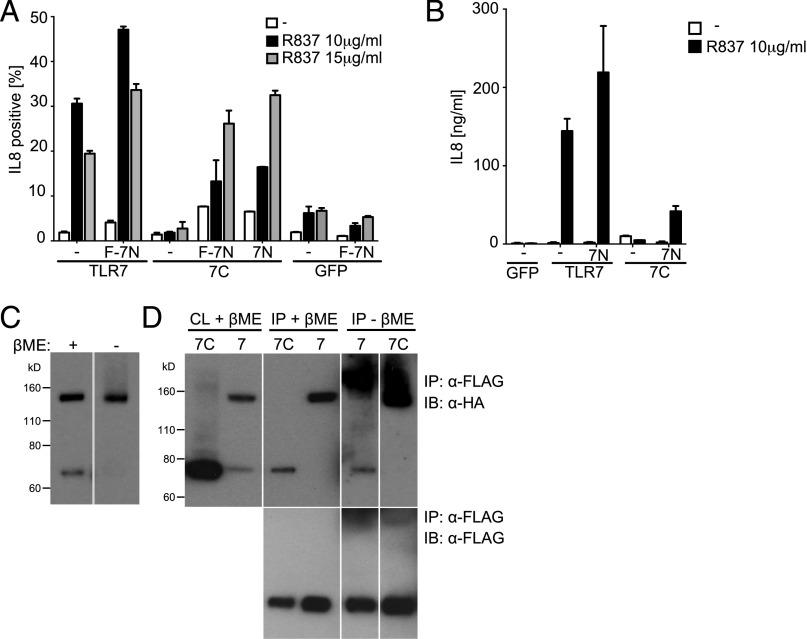
The N-terminal hTLR7 fragment associates with the C-terminal hTLR7 fragment and restores functional activity of the C-terminal hTLR7 fragment. (**A**) Intracellular FACS staining with anti–IL-8 Ab of THP-1 cells expressing the indicated constructs and stimulated for 14 h with 10 μg/ml R837 or 15 μg/ml R837 or not stimulated (white bars). Data are the percentage of positive cells ± SD of triplicates. Each cell line was tested for its ability to synthesize IL-8 upon coexpression of either the N-terminal hTLR7 fragment (7N) or the FLAG-tagged N-terminal hTLR7 fragment (F-7N). Staining in control samples not expressing 7N and F-7N is shown (-). (**B**) ELISA with anti–IL-8 Ab using tissue culture medium from THP-1 cells encoding the indicated constructs and stimulated for 24 h with R837 (10 μg/ml) or left unstimulated (white bar). Data are mean ± SD of triplicates. ELISA was performed in each cell line upon coexpression of the N-terminal hTLR7 fragment (7N). ELISA in control samples not expressing 7N is shown (-). (**C**) Lysates of THP-1 cells expressing full-length hTLR7 were incubated or not in loading buffer with 2-ME, and Western blot analysis with anti-HA Abs was performed. (**D**) THP-1 cells expressing FLAG-tagged N-terminal hTLR7 and encoding either HA-tagged full-length hTLR7 (7) or the HA-tagged C-terminal hTLR7 fragment (7C). Western blot of complete lysate (CL) with anti-HA–specific Ab in the presence of 2-ME (*upper far left panel*). Immunoprecipitation (IP) with anti-FLAG Ab, followed by Western blot with anti-HA Ab (*upper middle* and *right panels*). Immunoprecipitated samples were incubated in loading buffer, with or without 2-ME. Control samples, in which immunoprecipitation of the FLAG-tagged N-terminal hTLR7 was followed by Western blot with anti-FLAG Ab (*lower panels*). Representative of at least three independent experiments. 7C, THP-1 cells expressing C-terminal hTLR7 fragment; GFP, THP-1 cells expressing GFP; 7N, THP-1 cells expressing FLAG-tagged N-terminal hTLR7 fragment; THP-1, wild-type THP-1 cells; TLR7, THP-1 cells expressing full-length hTLR7.

We showed previously that, in THP-1 cells expressing full-length hTLR7, the C-terminal hTLR7 fragment is found in endosomal compartments and confers responsiveness to TLR7 agonists (14). Consistent with these findings, transfection of FLAG-tagged Unc93B1 reveals that the C-terminal hTLR7 fragment is associated with Unc93B1 (Supplemental Fig. 1). In contrast, transfection of a preprocessed C-terminal hTLR7 fragment failed to confer responsiveness to the TLR7 agonist R837 (14) (Fig. 1A). These results are consistent with the hypothesis that the hTLR7 N terminus provides essential functions. To test this hypothesis and to assess whether essential functions of the hTLR7 N terminus fragment could be provided in *trans*, we expressed a preprocessed hTLR7 C-terminal fragment in the presence or absence of a FLAG-tagged N terminus hTLR7 fragment (1–476) (Supplemental Fig. 2A) in THP-1 cells and tested them for responsiveness to the TLR7 agonist R837. Remarkably, we found that the N-terminal hTLR7 fragment could restore the activity of the C terminus TLR7 fragment, as measured by the percentage of IL-8^+^ cells (Fig. 1A) or by their secretion of IL-8 (Fig. 1B). The FLAG tag did not impact on the function of the N-terminal hTLR7 fragment because similar results were obtained with an untagged polypeptide (Fig. 1A). Introduction of the N-terminal hTLR7 fragment into THP-1 cells expressing the full-length hTLR7 also enhanced IL-8 production upon exposure to TLR7 agonists (Fig. 1A). In contrast, no response was seen in THP-1 cells expressing only the N-terminal hTLR7 fragment (Fig. 1A).

The above data suggested that the N- and C-terminal hTLR7 fragments might be able to associate with each other in *trans*. To test this possibility, we first examined whether the two fragments remain bound to each other after natural processing of hTLR7 by furin-like proprotein convertases. Indeed, in total cell lysates or in phagosomal fractions of THP-1 cells expressing full-length hTLR7, the receptor ran as a single band in nonreducing conditions (Fig. 1C, 1D). However, after the addition of 2-ME to reduce disulfide bonds, as used in our previous study (14), two distinct bands were observed (Fig. 1C, 1D). These results suggest that, after cleavage, the N- and C-terminal fragments of hTLR7 remain associated by means of disulfide bonding, even after trafficking to endosomal compartments. Notably, this could be recapitulated when the FLAG-tagged N-terminal hTLR7 fragment and the HA-tagged C-terminal hTLR7 fragment were expressed as separate polypeptides: upon immunoprecipitation with anti-FLAG Ab, followed by Western blotting for HA, a single band was detected under nonreducing conditions, which resolved into two bands of the appropriate molecular masses after the addition of reducing reagent (Fig. 1D). Interestingly, the FLAG-tagged hTLR7 N-terminal fragment also associated in a reducing-sensitive manner with HA-tagged full-length hTLR7 in the same assay (Fig. 1D).

We conclude that the N and C fragments of hTLR7 have intrinsic affinity for one another, permitting association and functional complementation in *trans*.

### The preprocessed C-terminal hTLR7 fragment mislocalizes to the cell surface unless directed to endosomes by the N terminus hTLR7 fragment

**FIGURE 2.**
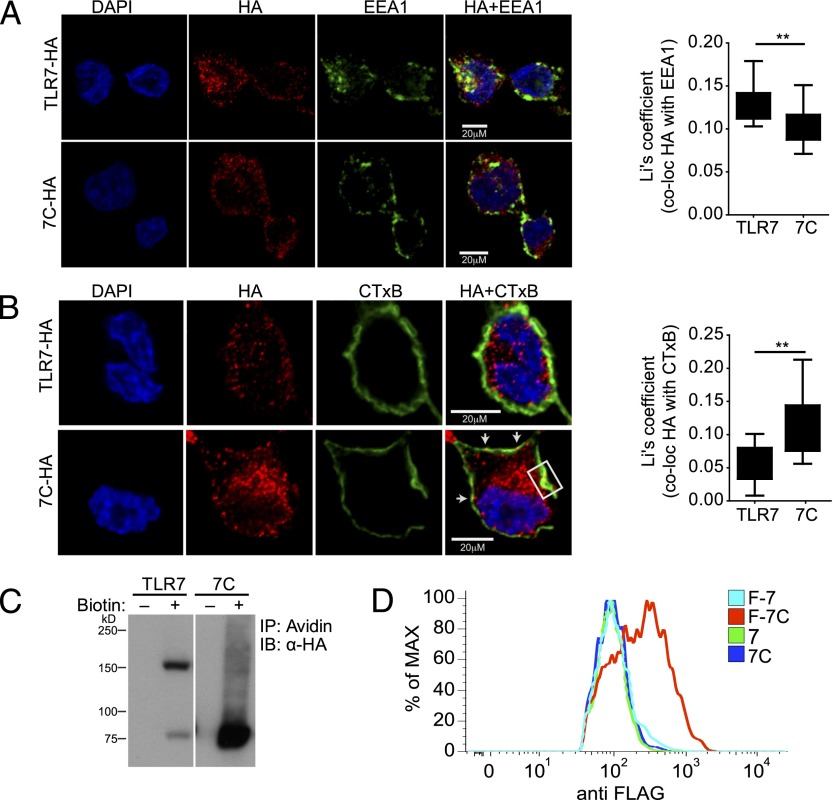
The C-terminal hTLR7 fragment localizes to the cell surface in the absence of the N-terminal hTLR7 fragment. (**A**) Colocalization of the HA-tagged C-terminal hTLR7 fragment (7C; red) with the early endosomal marker EEA1 (green) is reduced compared with the colocalization of full-length hTLR7 with EEA1 (*n* ≥ 16). (**B**) Colocalization of the HA-tagged C-terminal hTLR7 fragment (7C; red) with CTxB (green). White arrows and white box indicate areas of colocalization (*n* ≥ 9). In (A) and (B), THP-1 cells were fixed, permeabilized, blocked, and stained with the indicated Abs. Nuclear counterstain (DAPI) is shown in blue. Images were taken with a Zeiss inverted 780 confocal microscope. Quantification of colocalization was performed using Fiji. Li's coefficient was calculated to quantify the degree of colocalization. Data are represented as a box and whisker plot. Whiskers show minimum to maximum of all data. (**C**) Cell surface biotinylation of THP-1 cells expressing either HA-tagged full-length hTLR7 (TLR7) or the HA-tagged C-terminal hTLR7 fragment (7C), followed by immunoprecipitation using NeutrAvidin (Avidin) and Western blot analysis for HA tag. Control samples with no biotin are shown (-). (**D**) FACS staining with anti-FLAG Ab of THP-1 cells expressing the full-length hTLR7 and the C-terminal hTLR7 fragment with FLAG tag at the N terminus (F-7 and F-7C, respectively). Control staining with anti-FLAG Ab of THP-1 cells expressing the full-length hTLR7 and the C-terminal hTLR7 fragment without FLAG tag at the N terminus (7 and 7C) is also shown. Representative of at least three independent experiments. **p < 0.01, Student *t* test.

Confocal microscopy revealed that, in THP-1 cells expressing full-length hTLR7 tagged with an HA epitope at the C terminus, a portion of the label was correctly localized to endosomal compartments, where it colocalized with the early endosomal marker EEA1 (Fig. 2A). In contrast, colocalization with EEA1 was greatly decreased in cells expressing the HA-tagged preprocessed C-terminal hTLR7 fragment (Fig. 2A). Instead, the fragment accumulated at the plasma membrane, as revealed by colocalization with cholera toxin–labeled membrane (CTxB) patches (Fig. 2B). Mislocalization to the cell surface was confirmed using cell surface biotinylation (Fig. 2C) and flow cytometric staining (Fig. 2D). Importantly, we observed that a proportion of the full-length hTLR7 became surface accessible, as judged by the acquisition of biotin (Fig. 2C). However, it is likely that only a small proportion of hTLR7 is capable of reaching the cell surface, as shown by the lack of the N-terminal FLAG-tagged full-length hTLR7 with anti-FLAG Abs in nonpermeabilized THP-1 cells (Fig. 2D). These findings are consistent with earlier results describing surface biotinylation of TLR9 (21) and recently published findings demonstrating cell surface expression of mTLR7 (22).

**FIGURE 3.**
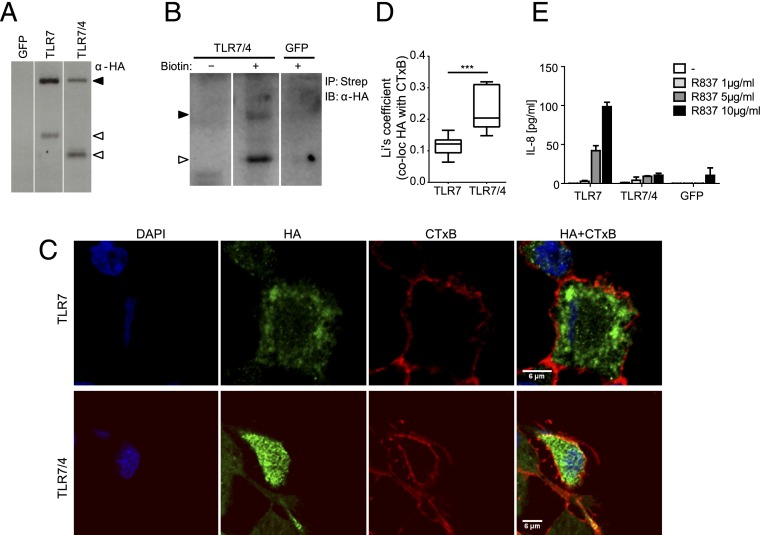
Cell surface–localized chimeric hTLR7/4 is functionally inactive. (**A**) Western blot of THP-1 cells expressing either chimeric hTLR7/4 (TLR7/4) or full-length hTLR7 (TLR7) with anti-HA Ab. Control samples of THP-1 cells expressing GFP are shown. Black arrowheads indicate full-length hTLR7 and hTLR7/4; white arrowheads indicate the C-terminal hTLR7 fragment (in cells expressing full-length hTLR7) and the C-terminal fragment of hTLR7/4 (in cells expressing chimeric TLR7/4 constructs). (**B**) Cell surface biotinylation of THP-1 cells expressing HA-tagged chimeric hTLR7/4 (TLR7/4), followed by immunoprecipitation using NeutrAvidin (Avidin) and Western blot analysis for HA tag. Black arrowhead indicates full-length hTLR7/4; white arrowhead indicates the C-terminal fragment of hTLR7/4. Control samples with no biotin (-) and with biotinylated GFP-expressing THP-1 cells (GFP) are shown. (**C**) Colocalization of HA-tagged hTLR7/4 (TLR7/4) and full-length hTLR7 (TLR7) (green) with the membrane marker CTxB (red). Nuclear counterstain (DAPI) is shown in blue. Cells were fixed, permeabilized, and stained with Abs. Images were taken with a Zeiss inverted 780 confocal microscope. (**D**) Enhanced colocalization of TLR7/4 with the membrane marker CTxB (*n* ≥ 7). Quantification of colocalization was performed using Fiji. Li's coefficient was calculated to quantify the degree of colocalization. Data are represented as a box and whisker plot. Whiskers show minimum to maximum of all data. (**E**) ELISA for IL-8 in THP-1 cells expressing the indicated constructs upon stimulation with the indicated concentrations of R837 for 24 h. Data are mean ± SD of triplicates. Representative of at least three independent experiments. ***p < 0.001, Student *t* test.

**FIGURE 4.**
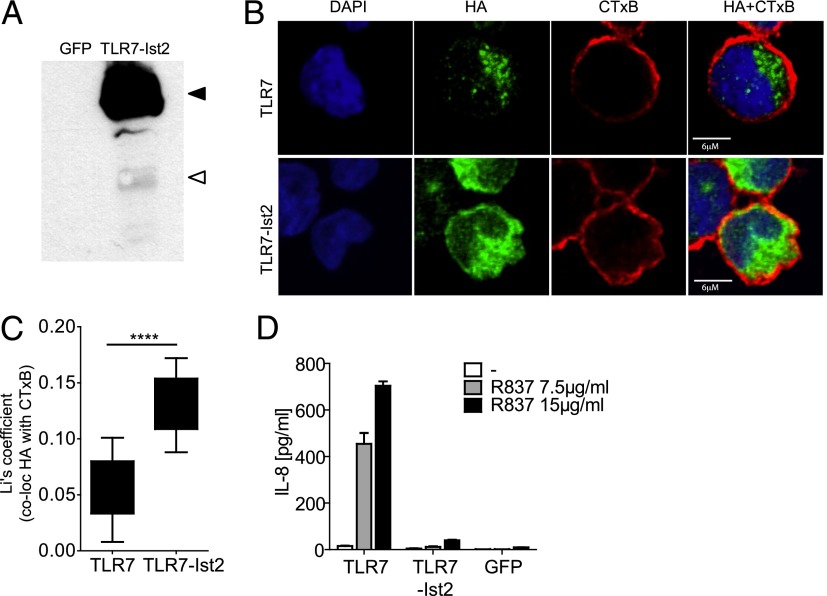
Cell surface–localized chimeric TLR7-Ist2 is functionally inactive. (**A**) Western blot of THP-1 cells expressing HA-tagged hTLR7-Ist2 (TLR7-Ist2) with anti-HA Ab. Control samples of THP-1 cells expressing GFP are shown. Black arrowhead indicates full-length hTLR7-Ist2; white arrowhead indicates the C-terminal fragment of hTLR7-Ist2. (**B**) Colocalization of HA-tagged hTLR7-Ist2 and full-length hTLR7 (green) with the membrane marker CTxB (red). Nuclear counterstain (DAPI) is shown in blue. Cells were fixed, permeabilized, and stained with Abs. Images were taken with a Zeiss inverted 780 confocal microscope. (**C**) Enhanced colocalization of TLR7-Ist2 with the membrane marker CTxB (*n* = 8 for TLR7-Ist2; *n* = 11 for TLR7). Quantification of colocalization was performed using Fiji. The Li coefficient was calculated to quantify the degree of colocalization. Data obtained are represented as a box and whisker plot. Whiskers show minimum to maximum of all data. (**D**) ELISA for IL-8 in THP-1 cells expressing the indicated constructs upon stimulation with the indicated concentrations of R837 for 24 h. Data are mean ± SD of triplicates. Representative of at least three independent experiments. ****p < 0.0001, Student *t* test.

To investigate whether mislocalization of the C-terminal hTLR7 fragment accounts for its lack of biological activity (Fig. 1), we engineered a chimeric hTLR7/4 molecule, consisting of hTLR7(Ecto) and the hTLR4 transmembrane and cytosolic domains (Fig. 3A). Confocal microscopy revealed surface localization of HA-tagged chimeric TLR7–TLR4 fusion protein (hereafter referred to as TLR7/4), as judged by its colocalization with the membrane marker CTxB (Fig. 3C, 3D) and by the acquisition of biotin (Fig. 3B). Importantly, although the hTLR7/4 chimeric molecule was processed into the correct shorter fragment (Fig. 3A), it failed to mediate responsiveness to the TLR7 agonist R837 (Fig. 3E). Similar results were obtained using full-length TLR7 fused at its C terminus to a fragment of the yeast protein Ist2, containing a plasma membrane-targeting motif (23, 24) (Fig. 4). Despite its correct processing into the correct shorter fragment (Fig. 4A), the chimeric TLR7-Ist2 protein localized to the cell surface, as judged by its colocalization with the membrane marker CTxB (Fig. 4B, 4C), and failed to mediate responsiveness to the TLR7 agonist R837 (Fig. 4D).

### The N-terminal hTLR7 fragment restores trafficking of the C-terminal hTLR7 fragment to endosomes

**FIGURE 5.**
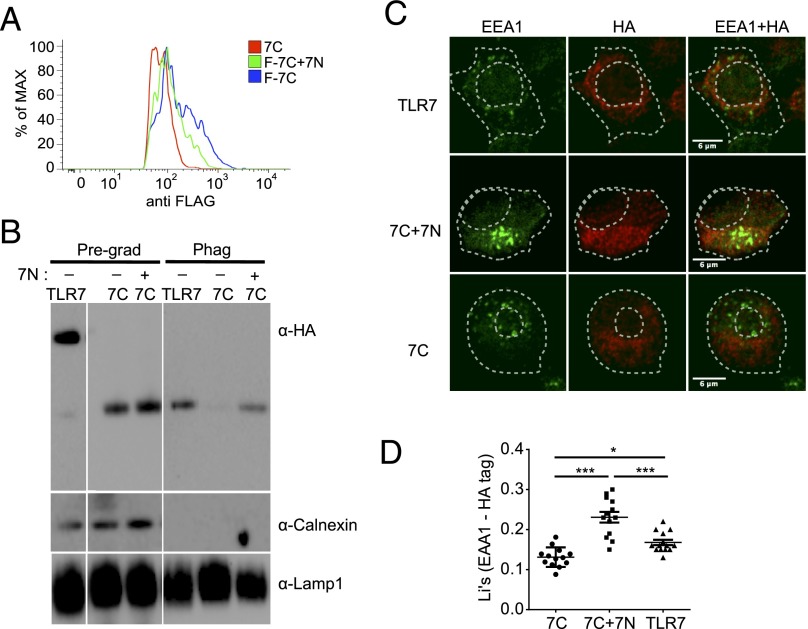
Expression of the N-terminal hTLR7 fragment targets the C-terminal hTLR7 fragment to endosomes. (**A**) Coexpression of the N-terminal hTLR7 fragment decreases cell surface localization of the C-terminal hTLR7 fragment. FACS staining with anti-FLAG Ab of THP-1 cells encoding the C-terminal hTLR7 fragment expressing (F-7C) or not expressing (7C) the FLAG tag at the N terminus. (**B**) Western blot with anti-HA, anti-Calnexin, and anti-Lamp1 Abs of phagosomes isolated from PMA-differentiated THP-1 cells expressing the indicated constructs. Coexpression (+) or lack of expression (-) of the FLAG-tagged N-terminal hTLR7 fragment is indicated. 7C, THP-1 cells expressing the C-terminal hTLR7 fragment; Phag, phagosomes; Pregrad, Western blot of the pregradient total lysate; TLR7, THP-1 cells expressing full-length hTLR7. (**C** and **D**) Colocalization of the HA-tagged C-terminal hTLR7 fragment (7C) with the early endosomal marker EEA1 is enhanced upon coexpression of the N-terminal hTLR7 fragment. HA tag staining is shown in red, and EEA1 staining is shown in green. THP-1 cells expressing the indicated constructs were differentiated with PMA for 24 h. Cells were fixed, permeabilized, blocked, and stained with the indicated Abs. Images were taken with a Zeiss inverted 780 confocal microscope. Cell membrane and nucleus positions are indicated by dashed lines. Quantification of colocalization was performed using Fiji. The Li coefficient was calculated to quantify the degree of colocalization. Blots show mean ± SE. 7C, HA-tagged C-terminal hTLR7 fragment; 7C + 7N, HA-tagged C-terminal hTLR7 fragment coexpressed with the FLAG-tagged N-terminal hTLR7 fragment; TLR7, full-length HA-tagged hTLR7. Endosomes were identified by the expression of the EEA1 protein (green). Representative of at least three independent experiments. *p < 0.1, ***p < 0.001, two-way ANOVA.

Because coexpression of the N-terminal hTLR7 fragment restored the functional activity of the C-terminal fragment in *trans* (Fig. 1A, 1B), we assessed whether the N-terminal hTLR7 fragment could restore trafficking of the C-terminal hTLR7 fragment to endosomes. Consistent with this hypothesis, coexpression of C- and N-terminal hTLR7 fragments in THP-1 cells decreased cell surface localization of the C-terminal fragment (Fig. 5A) and redirected the C-terminal hTLR7 fragment to phagosomes (Fig. 5B) or endosomes (Fig. 5C, 5D).

To assess further the role of the N-terminal hTLR7 fragment in restoring the functional activity of the C-terminal hTLR7 fragment in *trans*, we engineered a panel of N- and C-terminal hTLR7 fragments (Supplemental Fig. 2). Extra amino acid residues between leucine-rich repeat (LRR)14 and LRR15 (445–493) were shown to form a loop protruding from the LRRs that contain the TLR7 cleavage site (25). Consistent with this model, we showed previously, by mass spectrometry analysis, that the 75-kDa polypeptide derived from the processing of the full-length hTLR7 includes peptides mapped C-terminal of the region encompassing residues 445–493, which we showed encoding furin proprotein convertase cleavage sites (14).

To investigate the role of these residues in the interaction between the N- and C-terminal hTLR7 fragments, the N-terminal hTLR7 fragment truncated at residue 467 (7Ns; rather than the longer version truncated at residue 476) was expressed in THP-1 cells expressing in *trans* a range of C-terminal hTLR7 fragments (CtermG, CtermH, CtermI, CtermJ, and CtermK), either encoding or not encoding cysteine 475, which was known to play a key role in the formation of a disulfide bond between the C- and N-terminal fragments of mTLR7 (26). The results of these experiments showed that C-terminal hTLR7 fragments extended at the N terminus of residue 481 (fragments CtermJ, CtermI, CtermH, and CtermG) were surface accessible, as judged by the acquisition of biotin (Supplemental Fig. 2C), and were unable to render THP-1 cells responsive to the TLR7 agonist R837 (Supplemental Fig. 2B). We then showed that transfection of the shorter version of the N-terminal hTLR7 fragment (7Ns), unlike the results obtained with the longer N-terminal hTLR7 fragment, failed to enhance the biological activity of full-length hTLR7 and to restore the activity of the three different C terminus hTLR7 fragments (fragments CtermG, CtermH, and CtermI), as measured by the percentage of IL-8^+^ cells (Supplemental Fig. 2D).

Finally, in an attempt to identify mutations in the N terminus that abolish TLR7 activity, as shown by Iavarone et al. (27), as well as influence trafficking of hTLR7, we introduced mutations in the N-terminal part of hTLR7, in particular at positions 318 and 322 in the LRR14 domain upstream of the undefined loop, and as a control, inside the undefined loop (Supplemental Fig. 3A) (14). The results of these experiments, while showing that both mutations abolished the functional activity of the full-length hTLR7 molecule (Supplemental Fig. 3A), indicated that they also abolished the generation of the cleaved C terminal fragment (Supplemental Fig. 3B, 3C) without compromising trafficking to the endosomes (Supplemental Fig. 3D). These results are consistent with the possibility that mutations at positions 318 and 322 in the LRR14 domain upstream of the undefined loop abolish hTLR7 cleavage by furin-like protein convertases, rather than compromising the chaperoning function of the N-terminal hTLR7 fragment for hTLR7 into the endosomes.

**FIGURE 6.**
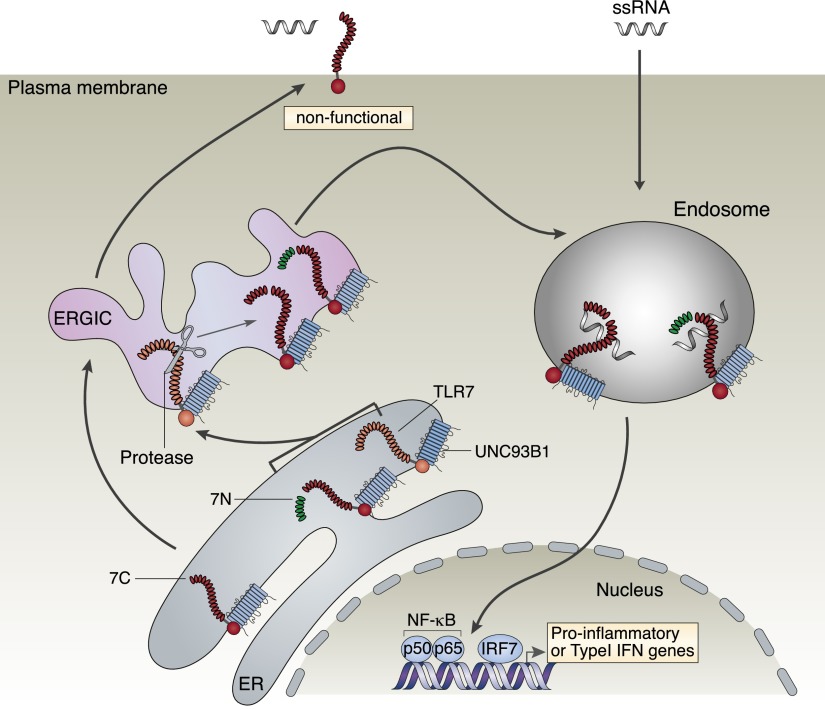
Schematic diagram illustrating trafficking of the different hTLR7 constructs. After cotranslational translocation into the ER, hTLR7 undergoes posttranslational proteolytic processing and traffics to endosomes. Only the processed receptors are functionally active. We showed previously that processing of hTLR7 could occur in a neutral pH compartment, defined in this figure as the ER–Golgi intermediate compartment (ERGIC). The full-length hTLR7 (TLR7) is shown in orange, whereas the amino-terminal (7N) and C-terminal (7C) fragments of hTLR7 are shown in green and red, respectively. UNC93B1, required for the correct folding of TLR7, is shown associated with the full-length hTLR7 and the C-terminal hTLR7 fragment. In the absence of the amino-terminal fragment, the C-terminal fragment of hTLR7 traffics to the cell surface and is functionally inactive. In contrast, coexpression of the amino-terminal fragment is required for proper trafficking of the hTLR7 C-terminal fragment to endosomes and restoration of functionality.

We conclude that the N-terminal fragment of hTLR7 provides essential trafficking information that allows redirection of the C terminus from the plasma membrane to endosomes (Fig. 6).

## Discussion

Correct trafficking and processing of TLR7 are critical for the ability of the receptor to act as a sensor of RNA viruses in the endolysosomal compartment. We previously found that TLR7 can be proteolytically processed at neutral pH by proprotein convertases of the furin family (14), suggesting that receptor processing could occur soon after biosynthesis in the ER, ER–Golgi intermediate compartment, or Golgi where those proteases can be found. The role of proprotein convertases of the furin family was recently extended to the processing of hTLR8 (28), demonstrating a more generalizable role of this family of proteases in endosomal hTLR-processing events. Early cleavage seems at odds with the recently solved crystal structure of hTLR8, which demonstrates a requirement for both the C- and N-terminal fragments in ligand binding (25). Similarly, the mTLR7 N-terminal fragment is required for the biological activity of mTLR7 (26), and it was shown that the N-terminal portion of TLR7 plays an important role in TLR7 function, in addition to its participation in ligand binding (27).

Consistent with these findings, it was shown that the N-terminal fragments of hTLR8 (28), TLR9 (29), and hTLR3 (30) are required for their biological activity. In this study, we demonstrate that the N-terminal hTLR7 portion plays a critical role beyond ligand binding in ensuring delivery of the C-terminal hTLR7 fragment to endosomal compartments; we also show that the two parts of hTLR7 can remain bound, even after cleavage, through disulfide bonding. Therefore, our data shed light on how the two halves of the processed receptor can still contribute to the formation of the optimal ssRNA binding site, despite being cleaved into separate polypeptides. Although the mechanisms by which the N-terminal hTLR7 fragment regulates trafficking of the C-terminal hTLR7 fragment remain unknown, our results are consistent with recently published results demonstrating that the N-terminal and C-terminal fragments of murine TLR9 bind each other and reconstitute a functional TLR9 receptor (26). We think it is unlikely that the N-terminal hTLR7 fragment acts merely by facilitating recruitment of the Unc93B1 chaperone, because the C-terminal fragment alone can associate with Unc93B1. However, it was shown previously that the adaptor protein AP-4 facilitates mTLR7 trafficking to endosomes (31); therefore, it is possible that the presence of the N-terminal fragment of hTLR7 is required for correct receptor interaction with adaptor proteins.

In the absence of the essential trafficking information provided by the N terminus, the processed C-terminal TLR7 fragment is misdirected to the plasma membrane. A recent study by Miyake’s group indicated that mTLR7 is expressed on the surface of ex vivo–purified splenic dendritic cells (DCs), whereas cell surface TLR7 expression on bone marrow conventional DCs, bone marrow plasmacytoid DCs, and B cells was much lower, suggesting the existence of a mechanism specifically controlling cell surface TLR7 expression (22). Consistent with these findings, we observed that a proportion of full-length hTLR7 reaches the cell surface, as indicated by the presence of biotinylated full-length hTLR7. Although we cannot rule out the possibility that surface accumulation of the C-terminal hTLR7 fragment may be due to its reduced internalization compared with full-length hTLR7, it is likely that, in THP-1 cells, only a small proportion of the total full-length hTLR7 reaches the cell surface, because N-terminal FLAG-tagged full-length hTLR7 failed to be detected at the cell surface, as defined by staining with anti-FLAG Ab.

Notably, we show that hTLR7 directed to the plasma membrane lacks biological activity. This suggests that localization of the C-terminal hTLR7 fragment in the endosomal compartment is required for receptor function, presumably because low pH is required for ligand binding and/or receptor signaling. Similarly, redirection of mTLR9 to the cell surface leads to loss of functional activity, because the receptor fails to be processed (23). In contrast, a chimeric mTLR9/TLR4 receptor is functionally active at the cell surface where it mediates responses to extracellular self-DNA (32). It was suggested that fusion of TLR9 with TLR4 allows the chimeric receptor to adopt a conformation that allows recruitment of MyD88 independently of proteolytic processing (23, 32).

In conclusion, our results highlight a novel function for the N terminus of hTLR7 by demonstrating its ability to direct trafficking of the processed receptor into endolysosomes. This previously unknown role for the N-terminal hTLR7 fragment ensures that processed hTLR7 receives the correct targeting instructions to reach the endosomal compartment, hence ensuring its biological activity. Endowing the cleaved fragment with the appropriate receptor-targeting signals might have evolved to ensure that any preprocessed hTLR7 that loses association with the N terminus is misdirected to the cell surface. The fact that this location is incompatible with receptor activity suggests that this form of regulation may have evolved to help prevent inadvertent responses to self-RNA and autoimmunity.

## Supplementary Material

Data Supplement
