# Supplementary material for: The Processed Amino-Terminal Fragment of Human TLR7 Acts as a Chaperone To Direct Human TLR7 into Endosomes
Source: J Immunol. 2015 Apr 27;194(11):5417–25. doi: 10.4049/jimmunol.1402703 (PMC4432728; doi:10.4049/jimmunol.1402703)
Supplement: Data Supplement [file JI_1402703.zip › JI_1402703_Supplemental_Material_1.pdf]

Supplementary Fig. S1

A

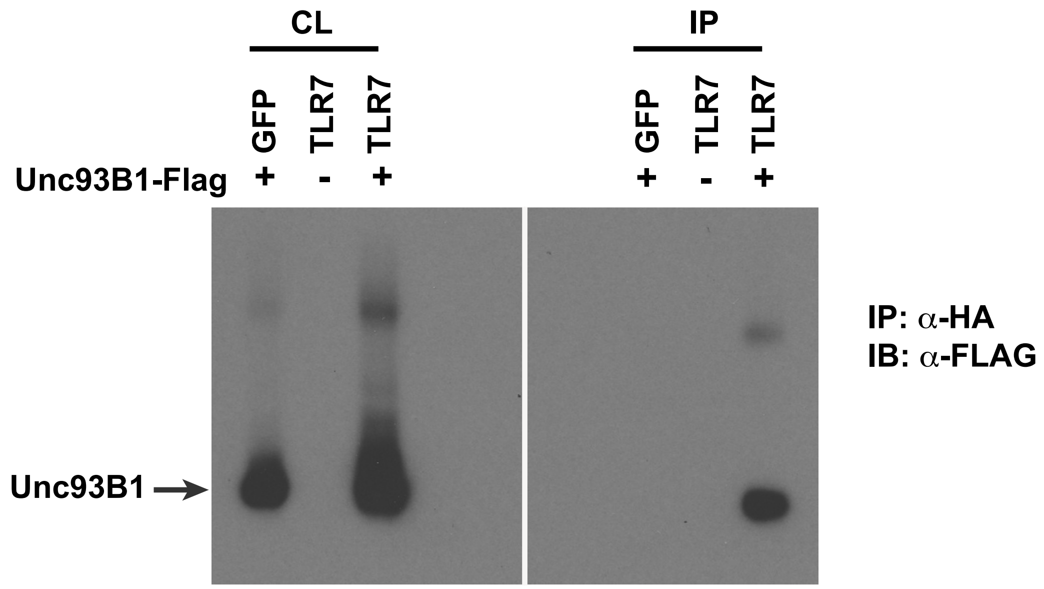

B

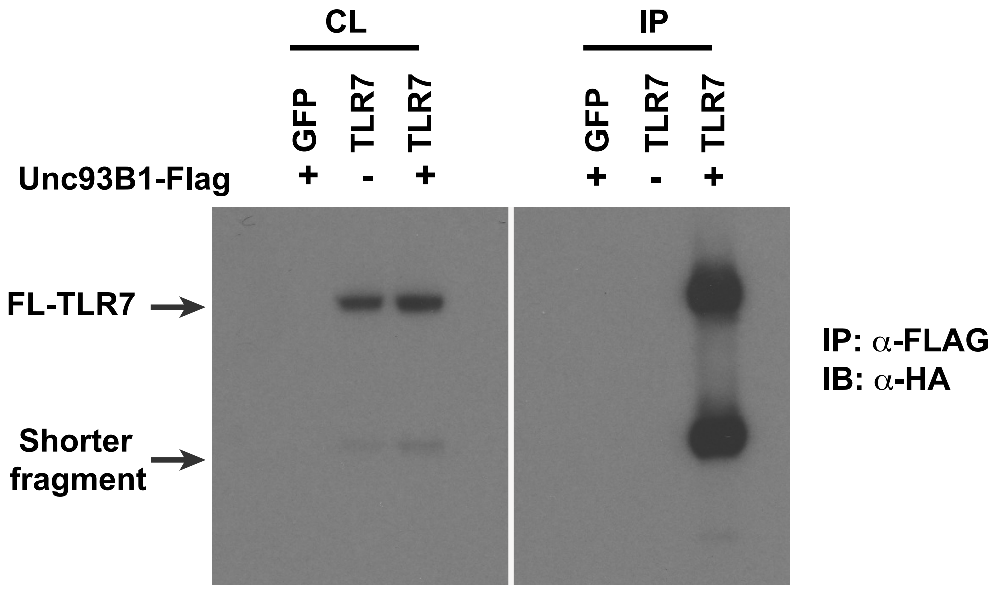

Supplementary Fig. S1: Unc93B1 associates with full length and C-terminal hTLR7 fragment

THP-1 cells expressing TLR7-HA alone, TLR7-HA+Unc93B1-FLAG or GFP+Unc93B1-FLAG.
(**A**) Left panel indicates western blot of complete lysate (CL) with anti FLAG specific Ab. Right panel indicates immunoprecipitation with anti HA Ab (IP) followed by a western blot with anti FLAG Ab (WB). (**B**) Left panel indicates western blot of complete lysate (CL) with anti HA Ab. Right panel indicates immunoprecipitation with anti FLAG Ab (IP) followed by a western blot with anti HA Ab (WB).

Supplementary Fig. S2

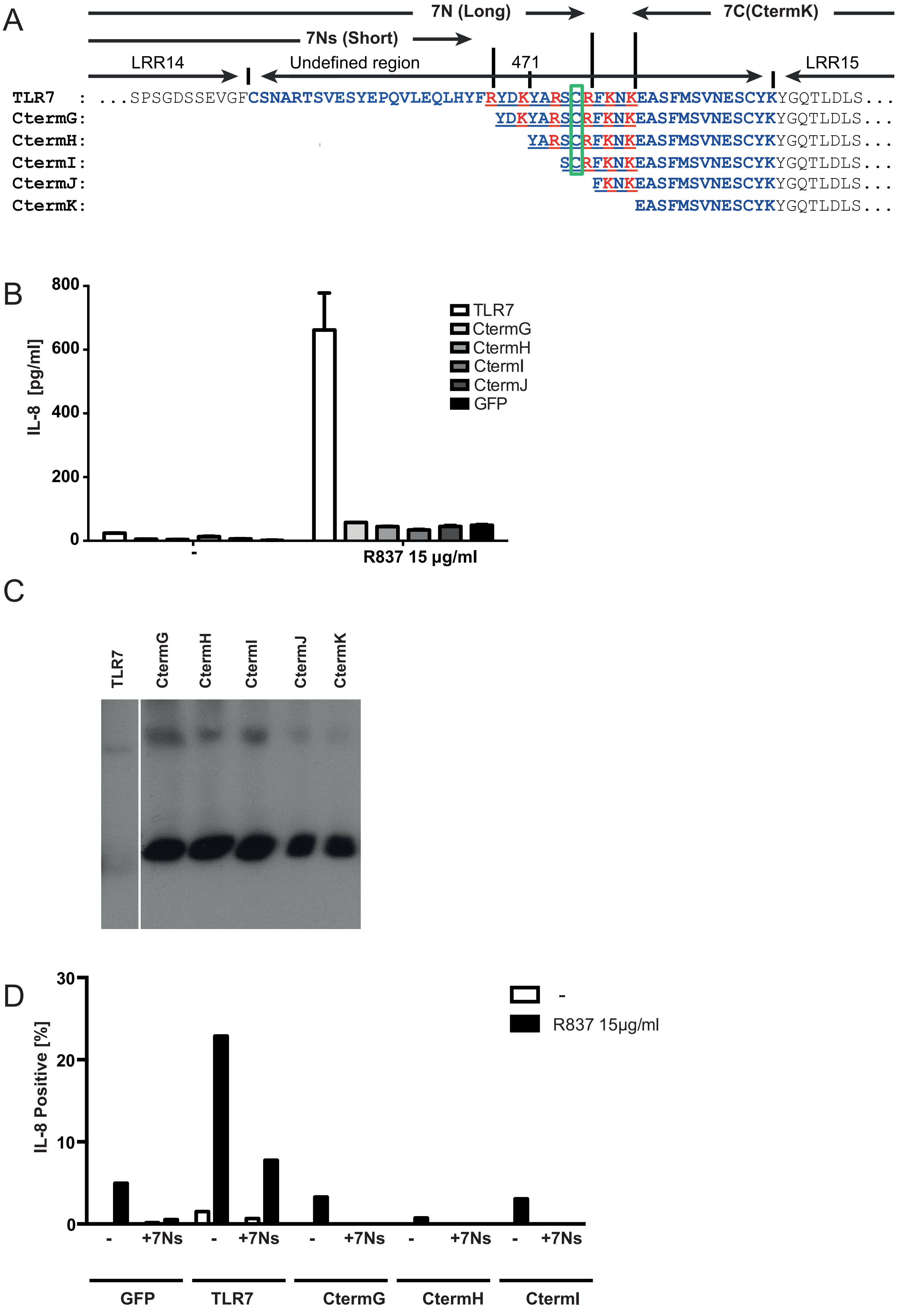

Supplementary Fig. S2: Co-expression of a shorter N-terminal hTLR7 fragment with the C-terminal hTLR7 fragment fails to restore functional activity of the C-terminal hTLR7 fragment

(A) Schematic diagram showing a range of N- and C- terminal hTLR7 fragments. The undefined region in the N-terminal hTLR7 fragment, in which cleavage occurs is shown in blue. A green box shows the location of a cysteine thought to play a role in disulfide bond formation between the N- and C-terminal fragments. In addition to the longer hTLR7 N-terminal fragment, corresponding to amino acids 1-476 of human Tlr7 (F-7N) which was used in the main figures of the manuscript, a shorter N-terminal fragment was made (7Ns), which lacked cysteine at position 475. We also engineered a range of C-terminal hTLR7 fragments CtermG to CtermJ, in addition to the CtermK used in all the main figures of the manuscript. (B) ELISA with anti IL-8 antibody using tissue culture medium from THP-1 cells encoding indicated constructs and stimulated for 24 hrs with or without R837 at concentration of 15μg/ml. Values indicate mean ± S.D. of triplicates. TLR7= THP-1 cells expressing full length hTLR7; CtermG, H, I, J= THP1 cells expressing TLR7 C-terminal fragments and GFP = THP-1 cells expressing GFP. (C) Cell-surface biotinylation of THP-1 cells expressing either HA-tagged full length hTLR7 (TLR7) or the indicated HA-tagged C-terminal hTLR7 fragments followed by immunoprecipitation using Neutravidin (Avidin) and Western Blot (WB) analysis for HA-tag. (D) Intracellular FACS staining with anti IL-8 antibody of THP-1 cells expressing indicated constructs and stimulated for 15 hrs in the presence (black bar) or absence of 15μg/ml R837. Values indicate % of positive cells. GFP = THP-1 cells expressing GFP, TLR7= THP-1 cells expressing full length hTLR7; CtermG, CtermH and CtermI = THP-1 cells expressing TLR7 C-terminal fragments; Each cell line was tested for its ability to synthesize IL-8 alone (-) or when the shorter hTLR7 fragment (7Ns) was co-expressed.

Supplementary Fig. S3

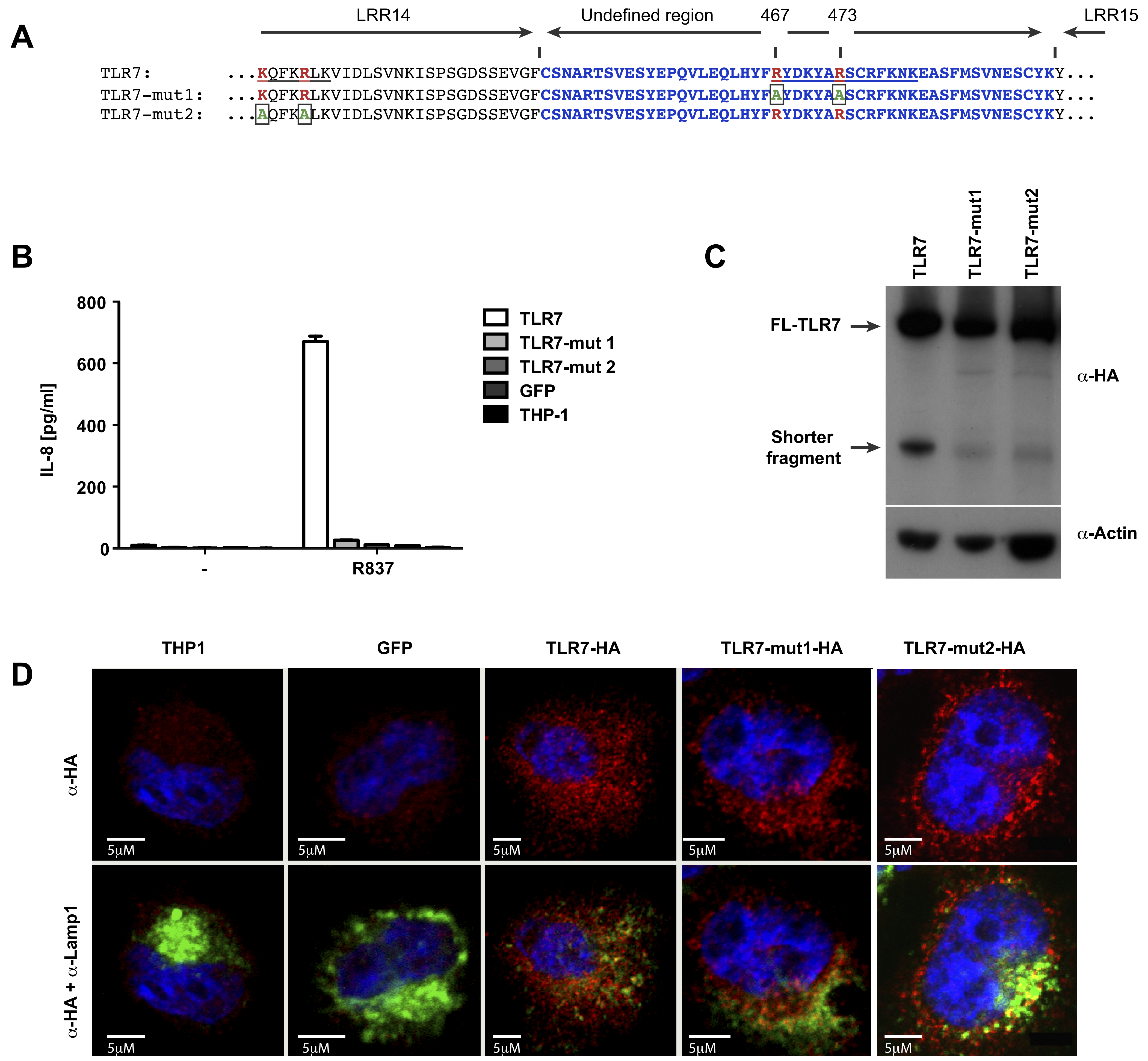

Supplementary Fig. S3: Mutating residues in the N-terminal region of TLR7 renders the receptor non-functional.

(**A**) Schematic diagram showing an alignment of regions of the ectodomain of TLR7 and TLR7 mutants. Underline indicates possible furin-like recognition motifs and the undefined region in which cleavage is thought to occur is depicted in blue. Boxes highlight the amino acid residues that were replaced by alanine in the mutants. (**B**) THP-1 cells expressing indicated constructs were stimulated with R837 (15µg/ml) for 24 hrs, IL-8 secretion was then measured by ELISA. (**C**) Anti-HA western blot of total cell lysate of THP-1 cells either expressing TLR7 or TLR7 mutants. (**D**) Confocal showing that both TLR7-mut1 and TLR7-mut2 co-localize with Lamp1. HA-tag staining is shown in red with the late endosome/lysosome marker Lamp1 shown in green. Nuclear counterstain (DAPI) is shown in blue. THP1 cells expressing the indicated constructs were PMA differentiated for 24 hours. Cells were then fixed, permeabilized, blocked and stained with the indicated antibodies. Images were taken with a Zeiss inverted 780 confocal microscope.

Table S1

Primer	Sequence: 5'--- 3' (restriction enzyme recognition site)
**Primers used for cloning**	
Fwd hTLR7HA	AACTTGATCACTGAGATCACCGGTAGGAGG (BclI)
Rev hTLR7HA	TGGTGTCGACGCTAGCTTTAGGCGTAGTCT (SalI)
A hTLR7/4 PCR Fwd	**AGTGTGATCTCCCTGGATCTGTACACCTGT**CAGATGAATAAGACCATCATTGGTGTGTCG[1]
A hTLR7/4 PCR Rev	CGACACACCAATGATGGTCTTATTCATCTG**ACAGGTGTACAGATCCAGGGAGATCACACT**[2]
mTLR7HA PCR Rev	TGGTCTCGAGGCTAGCTTTAGGCGTAGTCTGG (XhoI)
hTLR7 Mlu1 Fwd 2	AACTACGCGTAGGGCCACCATGGTGTTT (MluI)
hTLR7 linker Rev	TCTGGATCCTT*TGAACCTCCACCTCCAGAACCACCACCACCACTTCCTCCTCCTCC*GACCGTTTCCTTGAACACCTGACT[3] (BamHI)
Ist2 lenti Fwd	AACTTGATCAACGGCCCAGCAACAACAA (BclI)
Ist2 lenti Rev	ATATGCGGCCGCTTA*GGCGTAGTCTGGCACATCATAGGGGTA*GGATCCCAGCTTCTTCTTCAGCTTGTGCAG[4] (NotI)
TLR7Ist2 Bcl1 Fwd	AACTTGATCAAGGGCCACCATGGTGTT (BclI)
TLR7Ist2 SalI Rev	TGGTGTCGACTTAGGCGTAGTCTGGCACATC (SalI)
Flag Fwd	GACTACAAAGACGATGACGACAAGGCTAGATGGTTTCCTAAAACTC
Flag Rev	CTTGTCGTCATCGTCTTTGTAGTCTCTAGCCCCAAGGAGTTTGG
Bcl1kozak Fwd	TGATCACCACCATGGTGTTTCCAATGTGGACAC (Bcl1)
CtermK FLAG Fwd	GGCTGATCAGACTACAAAGACGATGACGACAAGGAGGCTTCTTTCATGTCTG (BclI)
TLR7 MluI Fwd	CGGACGCGTCCACCATGGTGTTTCCAATGTGG (MluI)
TLR7 Nterm Rev (Long)	GCCTGATCATTATCTGCAACTCCTTGCATAC (BclI)
TLR7 Nterm Rev (Short)	CCGTGATCATCTGAAATAATGTAATTGTTCC(BclI)
IRES Fwd	CGGGGATCCTGATCACGCCCCTCTCCCTCCCCCCCCCCTAACGTTACTGGCCGAAG (BclI)
IRES Rev	CCGGGATCCTTGTGGCCA (BamHI)
TLR7Mut1 Fwd	ACATTATTTCGCATATGATAAGTATGCAGCGAGTTGCAGATTC
TLR7Mut1 Rev	GAATCTGCAACTCGCTGCATACTTATCATATGCGAAATAATGT
TLR7Mut2 Fwd!	GCTAACCTCAGCATGTTTGCACAATTTAAAGCACTGAAAGTCATAGATC
TLR7Mut2 Rev	GATCTATGACTTTCAGTGCTTTAAATTGTGCAAACATGCTGAGGTTAGC

[1] Bold: Part of Fwd primer binding in TLR7 cDNA.
[2] Bold: Part of Rev primer binding in TLR7 cDNA.
[3] Italic: cDNA sequence coding for linker
[4] Italic: cDNA sequence coding for HA epitope
